# Supplementary material for: Are carbon emissions trading and green financial instruments synergistic? -Comprehensive quantitative research based on content analysis
Source: PLoS One. 2024 Mar 7;19(3):e0298601. doi: 10.1371/journal.pone.0298601 (PMC10919601; doi:10.1371/journal.pone.0298601)
Supplement: S1 Data — (ZIP) [file pone.0298601.s001.zip › Data/地级市Z_F工作报告.zip!/║■▒▒/╨ó╕╨╩╨/└┤╫╘╣1⁄2╓┌║┼í╛┬φ┐╦╩2╛▌═°í┐_2017.docx]

各位代表：

现在，我代表市人民政府向大会报告工作，请予审议，并请各位政协委员和列席人员提出意见。

一、过去五年工作回顾

市五届人大一次会议以来，在省委、省政府和市委的坚强领导下，在市人大、市政协的监督支持下，市政府同全市人民一道，沉着应对经济下行、旱涝灾害的双重考验，量质兼取壮实力，务实重行抓项目，精准服务强实体，倾心尽力惠民生，直面问题补短板，经济社会持续健康发展，全面小康建设迈出坚实步伐，较好完成了市五届人大一次会议确定的目标任务（见附件一），取得了“一个跨越、两个突破、三个提升、四个改善”的新成绩。

综合实力实现新跨越。坚持激活存量、扩大增量、壮大总量，主要经济指标居全省4—6位。地区生产总值每年以百亿级规模增长，2012年跨过千亿，预计2016年突破1500亿元，是2011年的1.6倍；地方一般公共预算收入2014年过百亿，2016年达到129.2亿元，是2011年的2.5倍；规模以上工业增加值超过720亿元，社会消费品零售总额超过880亿元，外贸出口突破10亿美元，分别是2011年的1.9倍、1.9倍和2.7倍；金融机构各项存贷款余额突破2000亿元和1000亿元。

改革创新实现新突破。以供给侧结构性改革激发活力，24项国家级、35项省级改革试点和其它各项改革有序推进，投资便利化改革成效明显，非行政许可审批全部取消，市级行政审批事项减少到263项，2967项市级权责清单对外公开，新登记市场主体年均增长16.9%。以创新创业释放新动能，制定“1+2+N”创新创业政策体系〔1〕，建立院士专家工作站57家、居全省第1位，高新技术企业达到190家、居全省第5位，孝感荣膺全国科技进步城市五连冠。以改革和市场办法破解融资、用地、人才难题，发行债券59亿元，主板上市企业4家、新三板〔2〕和四板〔3〕挂牌121家，向上争取、对内挖潜供地9.7万亩，乡村教师、乡村医生、乡村干部待遇提高，一批高素质人才扎根孝感。

区域统筹实现新突破。以“三项创建”〔4〕统揽城乡一体化建设，孝感获评国家园林城市、省级文明城市五连冠、省级卫生城市，全市获批省级文明城市4个、全国文明村镇9个，建设“三类新社区”〔5〕261个。建成高标准农田123万亩，扩挖塘堰10万多口，安全饮水惠及161.8万人，农村电网改造惠及39万户，公路总里程达1.6万公里，路网密度居全省第4位。以市区龙头经济、平原经济、山区经济“三大板块”统揽区域发展，市高新区跻身国家高新区、排名全省第4位，临空新城初具雏形、成为汉孝大临空三大组团之一，双峰山生态优势不断彰显，孝南获评中国生活用纸生产基地，汉川跻身全省县域经济第一方阵，应城获评中国最佳养生休闲旅游城市，云梦获批五个国家级农业示范创建试点，安陆成为全国蛋品加工基地和粮油机械之都，大悟打造华中新能源之都初具规模，孝昌基本建成苗木花卉百里走廊。

产业发展质效提升。坚持量质兼取，推动产业融合发展。规模以上工业企业1271家,居全省第5位,五年净增434家。7个产业集群列入省成长型产业集群，6个产业集聚区产值过百亿元，认定驰名商标13件、著名商标176件、湖北名牌78个、“三品一标”〔6〕701个。农产品加工产值1435亿元，居全省第4位，土地流转率达到37.5%，农机化率达到73.5%，建成省部级畜禽、水产健康养殖示范场84个和90个。限上商贸企业达到1107家，净增410家；商业网点达到12.8万个，净增2.2万个；网络商品零售额成倍增长，跨境电商达到96家；旅游人数突破2000万人次，综合收入达到132亿元。烟草、邮政、通信、石油、盐业等行业实现新发展。

项目投资质量提升。抢抓长江经济带、汉江生态经济带、大别山革命老区振兴发展等政策机遇，精准对接中省战略规划，滚动实施事关孝感发展全局的100个重大工程项目，储备“十三五”项目4299个、总投资3.8万亿元，累计争取中省项目资金68亿元，完成全社会固定资产投资7560亿元，年均增长24.4%，工业投资占比保持在45%以上，民间投资占比保持在65%以上。开展产业招商、二次招商，23家日资企业聚集高新区，48家中外500强企业落户孝感，累计引进省外资金3781.5亿元，实际利用外资15.4亿美元，分别年均增长19.8%和11.7%。

城镇建设品位提升。坚持全域理念完善城乡规划，完成《孝感市城市总体规划》和6个县市总体规划修编，常住人口城镇化率提升到54.9%。坚持建改并重拓展城市空间，孝感城区城建项目累计投资245.6亿元，建成区面积拓展到68.3平方公里，人口增加到65万人。新建改造城区道路40多条，综合整治背街小巷112条，新建老澴河桥4座，南城新区与主城区通道打通，东城新区起步区基本建成，新增公交线路9条。县市城区扩容提质，汉川、应城中等城市框架基本形成，云梦“三湖连河”、安陆河西新区、大悟高铁生态新城、孝昌“三河六岸”加快建设，辐射带动能力增强。坚持市场取向创新城市管理，大力推进“六大秩序”整治〔7〕，实行第三方检查考评，精细化管理水平不断提高，市容市貌更加干净整洁、文明有序。

生态环境明显改善。以规划引领“两型”社会建设，编制完成生态文明示范市建设规划，出台绿线、蓝线等5个生态专项规划。持续开展“绿满澴川”〔8〕行动，孝感城区改造、新建“六大主题公园”〔9〕，新增绿地450万平方米，全市创建省级生态镇7个、生态村114个，获批5个国家湿地公园，植树造林87.6万亩，建成7条绿色长廊，森林覆盖率提高到19％。实施最严格环保监管，持续开展污染综合防治，王母湖、野猪湖落实湖长制，全市新增城镇供水厂4座，新建改建城镇污水厂7座，城市生活垃圾无害化处理率、污水处理率分别达100%和95.9%，累计实施减排项目638个，主要污染物总量减排完成省定目标。

民生保障明显改善。五年累计向民生领域投入1015亿元，占财政总支出的77%。城镇和农村居民人均可支配收入分别从2011年的15888元、7029元，提高到28000元、13670元。城镇新增就业36.7万人，扶持困难群体就业4.1万人。“五项社保” 〔10〕参保突破850万人次，养老保险基本实现全覆盖，城乡居民医疗保险完成整合，医疗保险、医疗救助、重特大疾病救助全面衔接。投入18亿元新改扩建民政公共服务设施800多个，社会救助标准大幅提高，发放救助资金35亿元，常年救助31万人次。建成保障性安居房18.4万套，改造农村危房3.5万户，归集公积金68.1亿元。持续开展脱贫攻坚，五年脱贫27.2万人。投入30.9亿元实施学前和义务教育项目4242个，新增公办幼儿园112所，新建中小学33所，建成装备标准化学校553所，改扩建校舍178万平方米，7个县市区全部通过义务教育均衡发展国家认定，留守儿童关爱经验全国推广，市县城区全面推行中小学划片就近入学，5所中职学校获评国家和省示范职业学校，湖北工程学院、湖北职业技术学院办学实力增强。投入9亿元实施卫生基础设施项目889个，新增医疗用房39.2万平方米，县级公立医院综合改革实现全覆盖，出生人口性别比五年下降15个单位值。“五项惠老政策”〔11〕全面落实，累计发放“敬老红包”过亿元，“四项助残行动”〔12〕惠及残疾人10.5万人次。全市“三馆一站”〔13〕免费开放，每年举办中华孝文化旅游节，国家级非遗名录达6项，建成全民健身工程2175处。

社会治理明显改善。基层治理体系不断完善，村（社区）综合服务中心实现全覆盖，“共治+自治”的格局加快形成。信访维稳体系不断完善，推进立体化治安防控，推行“一村一警”下沉警力，城乡网格化管理实现全覆盖，网上信访、法治信访成为解决群众诉求的新常态，平安孝感建设走在全省前列。安全风险防控体系不断完善，生产安全事故死亡人数、亿元GDP事故死亡率五年下降10.5%和36%，市县产品质量监测平台全面整合，食品药品三级监管网络全部建成，突发事件应急处置及时有效。各项事业全面均衡推进，孝感荣获全国双拥模范城七连冠、全国未成年人思想道德建设先进城市，人防、老龄、地方志获评全国先进集体，民族宗教、外事侨务、对台工作迈上新台阶，统计、调查、水文、气象、物价、保密、档案、驻外等工作取得新成绩。

行政效能明显改善。坚持依法行政，自觉接受市人大、市政协监督，累计办理人大代表建议517件、政协委员提案1213件；“六五”普法全面完成，学法考法、法律顾问、规范性文件法制审查、集体议事决策等制度健全完善，政府立法和协助人大立法工作有序推进。提高执行力落实力，建立健全重难点突破、主责清单管理、督查约谈问责、“一线工作法”等落实机制，以市长信箱常年收集、及时解决民生问题，形成层层抓落实的制度链条。提升服务效能，市县乡村四级政务服务体系全面建成，下沉服务事项300项，群众和企业办事更加便利。坚持廉洁从政，扎实开展党的群众路线教育实践活动、“三严三实”专题教育和“两学一做”学习教育，“四风”整治〔14〕成效明显，主体责任全面落实，纠风正纪、执法监察、治庸问责、审计监督、国资监管、项目整体打包招标等制度笼子越扎越紧。

刚刚过去的2016年，改革发展任务繁重，实体经济困难重重，洪涝灾害历史罕见，全市上下主动作为，知难而进，稳住经济社会发展大局，实现了“十三五”良好开局（见附件二）。一年来，我们全力以赴抗灾救灾，万众一心抗击超历史、超98特大洪涝灾害，广大干部群众与7000多名部队官兵、公安干警日夜奋战在抗洪抢险一线，609个工作队对口帮扶连片重灾村，积极落实“七补”“六抢”措施〔15〕，粮食总产稳定在44亿斤以上，2401户因灾倒房全部恢复重建。我们精准帮扶实体经济，出台企业降成本28条、房地产去库存19条、电子商务发展22条，开展“优质服务办成事”“千名干部进千企”活动，组织惠企政策解读13场次，全面推开“营改增”〔16〕，为中小企业减税降费7.9亿元；综合运用“集合贷”“增信贷”、融资担保金、续贷周转金等手段，为企业融资46亿元。我们深入推进改革创新，以供给侧结构性改革为主线，有序推进投融资体制、户籍制度、不动产登记、集体林权、粮食流通、国有农场林场、农村土地确权登记颁证、农村产权交易、供销社综合改革、机关事业单位养老保险、公务用车等各项改革；兑现“双创”奖励扶持资金1200万元，发放科技创新券1585万元、创业担保贷款2.1亿元，扶持带动创业就业1.45万人，新申请发明专利1613件，孝感通过国家知识产权试点城市考核验收。我们千方百计扩大投资，积极开展对外招商，引进投资过亿元项目96个、过10亿元项目12个；组织开展重大项目“开工周、开工月”活动，新开工投资过5000万元项目216个；策划申报44个灾后重建项目，总投资561.9亿元。我们持续用力改善环境，以中省环保督察为契机，铁拳治理环保突出问题，采取城区禁鞭、锅炉禁煤、秸秆禁烧、扬尘治理、油气回收、黄标车淘汰、工业污染源减排等举措，强力整治大气污染，PM10、PM2.5平均浓度降幅和空气优良天数增幅均居全省第一；建成市垃圾综合处理场和25座乡镇垃圾中转站，启动184个美丽乡村建设，创建省级森林城镇4个、绿色示范乡村147个。我们尽心尽力改善民生，聚焦聚力精准脱贫，统筹推进易地扶贫搬迁，9万贫困人口脱贫、179个贫困村出列；百件实事全部完成年度任务，176所学校“全面改薄”〔17〕，两孩政策全面实施，成功举办市五运会，居民基本医疗保险和大病保险同步结算；市游泳馆对外开放，文化中心、市民之家基本建成，武孝城铁建成通车；推行安全生产“双随机”制度〔18〕，建立“互联网+法律援助”平台，划定4021个城乡“小网格”服务民生、化解矛盾，社会保持和谐稳定。

各位代表，回顾过去五年的工作，我们深深体会到：必须坚持党的领导，自觉维护市委权威，凝聚合力，增强政府执行力，打基础、利长远，一张蓝图绘到底。必须坚持第一要务，以第一力度抓项目、强实体、促发展，精准施策稳增长，保持定力促转型，远谋近施做大总量提升质量。必须坚持人民至上，保基本、兜底线、促公平，全民共治、共建共享，让发展成果惠及广大人民群众。必须坚持改革创新，转换思维，搭建平台，营造环境，向改革要红利、向创新要动力、向开放要空间，激发内生动力和市场活力。必须坚持真抓实干，勇于担当、立说立行，在一线服务群众、帮扶企业，在一线发现问题、破解难题，以重难点突破带动全局。必须坚持清正廉洁，守纪律、讲规矩、转作风，用制度规范权力、管好班子、带好队伍，以良好形象取信于民。

五年的成绩来之不易。这是省委、省政府和市委正确领导的结果，是全市人民共同奋斗的结果，是社会各界大力支持的结果。在此，我代表市人民政府，向辛勤工作在各行各业的广大干部群众，向各民主党派、工商联、人民团体、各界人士和离退休老同志，向驻孝部队、武警官兵和中省单位，向所有关心支持孝感发展的同志们、朋友们，表示衷心的感谢和崇高的敬意！

在总结成绩和经验的同时，也要看到政府工作中的困难和问题。主要是：经济总量偏小，产业层次不高、结构不优，县域经济发展不均衡，尤其在开放型经济、“四新经济”〔19〕和重大项目支撑方面，还存在较大差距；全面深化改革和推进创新创业的任务繁重，制约发展的体制机制障碍还有待突破；公共服务、农田水利等基础设施欠账较多，民生保障与人民群众的期盼还有差距；少数公务员工作作风不适应新常态的要求，不敢为、不善为、不想为的现象依然存在。对于这些问题，我们将在今后的工作中认真加以解决。

二、今后五年目标任务

今后五年，是全面建成小康社会的决战期，也是孝感加快发展的战略机遇期。刚刚召开的市第六次党代会，科学研判了我市面临的机遇挑战，描绘了孝感全面小康的美好蓝图。把党代会确立的目标任务落到实处是政府的职责所在，让人民生活更加美好是政府的使命担当。今后五年，政府工作的总体要求是：高举中国特色社会主义伟大旗帜，以马克思列宁主义、毛泽东思想、邓小平理论、“三个代表”重要思想、科学发展观为指导，全面贯彻落实党的十八大、十八届三中四中五中六中全会和习近平总书记系列重要讲话精神，统筹推进“五位一体”总体布局，协调推进“四个全面”战略布局，有效把握引领经济新常态，坚定不移践行五大发展理念，大力推进“五个跨越”，加快建设“五个城市”〔20〕，奋力开创孝感第三次创业新局面，确保全面建成小康社会。

今后五年，我们将按照市第六次党代会确定的奋斗目标，加快建设“五个城市”，经济社会保持持续健康发展，主要经济指标总量居全省前列，增幅高于全国、全省、武汉城市圈平均水平（见附件三）。一是加快建设经济强市，不断提升综合实力。坚持大产业、大市场、大融合理念，推动新型工业、现代农业、新兴服务业融合发展，实施中国制造2025孝感行动，形成食品饮料、纺织服装、盐磷化工和战略性新兴产业等4个千亿工业支柱产业；完善现代农业产业体系、生产体系和经营体系，农产品加工产值突破2100亿元；打造区域性现代服务业中心，社会消费品零售总额突破1400亿元。二是加快建设鄂豫省际区域性中心城市，不断提升辐射带动力。坚持“全域孝感”理念，推动“六大组团”〔21〕融合壮大，孝感中心城市建成区面积超100平方公里、人口超100万人，辐射带动能力进一步增强。建成5个县市中等城市、30个特色小镇、100个新型农村社区，形成以孝感中心城市为龙头、县市城区为骨干、乡镇集镇为支撑的市域城镇体系。三是加快建设中华孝文化名城，不断提升文化软实力。坚持城市文明与特色文化相融并进，弘扬社会主义核心价值观，发扬“至孝至诚、图强图新”的孝感精神，修复修建文昌阁等一批遗址遗迹，新建一批孝文化景观和标志性建筑，打造孝文化一条街和主题公园。促进孝文化与红色文化、楚文化、李白文化及民俗文化的深度融合，整合资源、市县联动举办中华孝文化旅游节。发展壮大文化事业和文化产业，全面建成“文化小康”。以“四孝”品牌〔22〕传承孝文化，孝感建成全国文明城市，县市争创1个国家级、2个省级文明城市。四是加快建设创新城市，不断提升内生动力。坚持由要素驱动向创新驱动转变，完善“1+2+N”政策体系，建成“1+3+7”双创载体〔23〕，深入实施“槐荫人才计划”〔24〕“大学生创业资助专项计划”和百名领军人才、百个创新团队、百名科技之星“培育工程”，新增国家级创新平台5家，争创全国创新创业示范基地，建成国家级创业型城市和国家知识产权示范城市，建设国家创新型城市。五是加快建设人民幸福城市，不断提升群众幸福感知力。坚持以人民为中心，把更多财力向民生倾斜，居民人均可支配收入达到3万元，基本养老、城乡救助和福利水平明显提高，城乡一体、全民享有的社保体系健全完善，教育、医疗、文体、就业、住房等民生兜底能力全面提升，让发展成果更多更公平地惠及全市人民。

今后五年，我们将按照市第六次党代会的总体安排，把目标任务具体化、项目化，着力抓好十项重点工作。

1.打赢精准脱贫攻坚战。把脱贫攻坚作为重大政治任务、重大民生工程和重大发展机遇，到2018年底，完成1.52万户易地搬迁,全市24.92万贫困人口全部脱贫，444个贫困村出列，2个贫困县“摘帽”,以精准脱贫补齐“三农”发展短板。

2.深化供给侧结构性改革。按照中央和省里要求，认真落实“三去一降一补”〔25〕改革任务，新型城镇化、知识产权等国家试点形成“孝感经验”，更多中省改革试点在孝感落地开花结果。坚持出台方案与落实方案并重，深化经济体制、行政体制、投融资体制、农村产权制度、生态文明体制等重点领域和关键环节的改革，以改革破难题、促发展。

3.拓展开放融合平台。主动融入一带一路、长江经济带、汉江生态经济带和武汉城市圈，积极对接武汉综合保税区和内陆自贸区政策，配套承接国家航天产业基地项目，市高新区建成国内重要的军民融合产业示范基地，市临空区建设保税物流中心，推动25个优势特色产业集聚区优化布局、转型升级，形成现代制造、都市产业、绿色产业聚集发展轴。

4.发展壮大“四新经济”。落实省政府《关于加快发展新经济的若干意见》，构建人才、技术、资金、创意、信息等各种生产要素向新经济自由流动的体制机制，引导战略性新兴产业成长壮大，鼓励发展“互联网+”、物联网、云计算、电子商务等新业态，在智能制造、个性化定制生产、数字工厂和一二三产业融合等方面，扶持一批“四新经济”，培育壮大新动能。

5.建设华中地区旅游目的地。提升旅游通达能力，实现市内4A级景区旅游专线互联互通，打通双峰山至木兰湖、观音湖至黄陂清凉寨、大悟至黄陂蔡店、观音湖至河南新县旅游通道，全面融入大别山区域和武汉城市圈旅游。提升景区综合服务水平，开展A级景区和旅游名镇名村名街创建，新创5个4A级景区，推进文化与旅游深度融合，凸显孝文化体验、生态康养、红色教育、楚文化游览等特色，唱响“孝感天下、情润山水”旅游品牌。

6.建设大数据孝感。深入开展信息惠民国家试点城市建设，实施“宽带乡村”国家示范工程〔26〕，市县城区重要公共场所实现免费无线网络全覆盖，完善大数据产业链，建成智慧政务服务、智慧交通出行平台，大力发展远程医疗会诊、远程教育、食品药品远程在线监控，形成完备的智慧政务、智慧医疗、智慧教育、智慧养老、智慧交通和工业智造体系，建成光网城市。

7.完善大交通体系。加快形成内外连通、全域通达的快速轨道交通网络，汉十高铁建成通车，启动建设孝感西站至武汉直通线、孝感东站至北站城际铁路、武汉—荆门城际铁路汉川段、随州—安庆铁路大悟段，立项申报汉十高铁云梦—应城联络线。完善“三横三纵三环六网”市域交通体系〔27〕，五年新增公路4000公里，建成对接武汉、连通7个县市区的高速公路循环线；建成316、107新外迁线，形成孝感城区一级公路外环线；构筑澴川路—316复线—宝成路—长兴三路—107国道城区内环线；打通城区内环节点，完善城区公共交通体系和微循环路网。

8.建设美丽宜居孝感。全面实施生态立市战略，持续开展生态文明创建，推行海绵城市和地下综合管廊建设，把临空区建成全国地下综合管廊和海绵城市示范区。加强国家湿地公园和生态功能区保护，建成600个美丽乡村，城镇公共绿地面积达到28平方公里，森林覆盖率达到20%，水、大气、土壤质量明显改善，孝感建成国家卫生城市、生态园林城市、森林城市和环保模范城市，所有县市争创国家园林城市、省级森林城市。

9. 共享健康、教育、养老均等化服务。落实“健康中国2030”规划纲要，建设东城新区医疗服务中心，建成“15分钟基本医疗服务圈和健身休闲圈”，办好医疗共同体，支持医养结合，发展健康产业，实施全民健身计划，促进全民健身和全民健康深度融合，建设健康孝感。发展普惠性学前教育，提升义务教育均衡发展水平，关心支持特殊教育，普及高中阶段教育，推进职业教育发展，鼓励社会力量办学，全市实施重大教育项目96个，基本消除城区学校大班额现象，城乡教育布局更加均衡合理。关心下一代健康成长，留守儿童实现“六有关爱”〔28〕，所有老人享有“六有”生活〔29〕，让孩子们快乐成长、老年人颐养天年。

10.建设法治平安孝感。加强城乡建设管理、环境保护、历史文化保护等领域立法，压实依法行政“第一责任”，构筑市县乡村一体化法治平台，深入推进综合执法、文明执法、公正执法，建设法治政府。建成覆盖全社会的征信系统，构建食品药品社会共治体系，织密织牢防控“八张网络”〔30〕，完善立体化社会治安防控体系，健全安全生产责任和突发事件应急处置体系，建立资源共享、快速高效的公共安全保障网络，全面提升人民群众的安全感。

三、2017年工作安排

2017年是新一届政府的开局之年，也是实施“十三五”规划的重要一年。我们将围绕年度预期目标（见附件四），全力抓好十项工作。

（一）全力推进中国制造2025孝感行动落地见效。推动工业转型升级。编制《工业企业技术改造指导目录》，运用现代装备和信息技术改造提升传统产业。围绕新一代信息技术、智能装备、新能源汽车、生物医药、新材料、节能环保装备等六大领域，组织实施十大重点示范项目，推动战略性新兴产业升级发展。开展智能工厂、数字化车间建设试点，加快“两化”融合试点建设。积极对接武汉国家航天产业基地，推进军民融合深度发展,军民融合产值过200亿元。助推企业提质增效。以提高质量和竞争力为中心，做优存量、做大增量，优化整合产业集聚区，销售过亿元企业达600家、过10亿元企业34家，新增产值过百亿集聚区1个，新增驰名商标2件、著名商标20件。精准对接服务企业。支持各级园区开展管理和运营机制创新，为企业提供全方位服务。扎实开展政策宣讲、金融服务、产销对接等活动，瞄准政策落实中的细节难题，瞄准企业反映强烈的突出问题，定向、定期、精准落实降成本28条优惠政策，帮扶企业保障生产要素、降低成本，为企业成长壮大营造良好环境。

（二）全力推进现代农业融合转型。推进农业供给侧结构性改革。大力发展规模农业、科技农业、品牌农业、观光农业、安全农业，推动立体种养、绿色有机循环农业连接，全面落实从农田到餐桌全环节食品安全监管，增加绿色优质农产品供给，“一高三新”〔31〕覆盖率达50%，农产品质量安全率达98%以上，新增省部级水产健康养殖示范场33家、无公害基地15个。推动现代农业融合发展。培育新型职业农民，扶持壮大家庭农场、专业大户、农民合作社、农业产业化龙头企业等新经济主体，推动纺织、粮油、食品、纸品木制品产业加快发展，农产品加工产值增长9%以上。促进农产品营销线上线下融合，建设一批标准化“互联网+”现代农业示范基地。夯实现代农业基础。完善防汛应急和灾害预警预报体系，建设防洪保安、闸站更新改造、中小河流治理、抗旱应急水源、重点区域排涝等九大水利工程，在主汛期前全面提升防汛抗旱减灾能力。完成第三次全国农业普查入户登记工作，加强农田修复和保护，坚守耕地红线，建设标准农田56万亩。抓好脱贫攻坚。集中财力办大事，整合“五个一批”〔32〕政策资金，新建通村公路200公里，村道加宽400公里，改造农村危房6000户，全面实施整村推进、产业扶贫、易地搬迁，统筹推进脱贫攻坚与大别山试验区建设，确保9.85万贫困人口稳定脱贫、167个贫困村出列。

（三）全力推进新兴服务业增量提质。改造提升商贸物流业。启动建设东城新区邻里中心。推进县市城区商业网点改造和放心早餐店建设，加快农贸市场和社区菜市场标准化建设,大力发展农村消费品直营连锁网络和专业市场。培育发展一批现代物流园区。新增限上单位50家。突破性发展适应“四新经济”的新兴服务业。围绕新型工业化、新型城镇化、农业现代化、城乡生态化，培育和壮大研发设计、服务外包、商务咨询、节能环保等新兴服务业态，鼓励发展养老托幼、社区医疗、家政服务、物业管理等社区现代服务业。支持电子商务加快发展，电子商务交易额增长35%以上。深入实施外贸“三项”工程〔33〕，新增出口企业30家、过千万美元骨干企业5家。壮大现代金融业。开展新兴金融业培育行动，优化金融保险发展环境，加强多层次资本市场建设，防范系统性金融风险，深化银企对接，每个银行、保险业机构至少创新1个有本地特色的金融产品，力争新增贷款100亿元。整合发展旅游产业。完善旅游基础设施，开展旅游标准化创建，促进文化与旅游业融合、脱贫攻坚与乡村旅游结合，打造乡村生态文化旅游示范带，推进全域旅游。组建旅游发展集团，规划建设旅游集散中心，整合打造精品线路，全年接待游客2300万人次，实现综合收入150亿元。促进房地产市场平稳健康发展。支持合理自购住房，严控投资投机性购房，因城因地施策，减少房地产库存。出台小区物业综合治理意见，改造棚户区1.85万户。新增公积金实缴人数1万人、归集公积金15亿元。

（四）全力推进改革创新深度发力。向改革要活力。落实好中省各项重大改革举措，扎实推进政府层面的重点领域、重要试点改革任务。深化供给侧结构性改革，深入推进“三去一降一补”，落实降费减负政策惠企利民。深化“放管服”改革，完成承担行政职能事业单位改革试点，全面推行企业“五证合一、一照一码”和个体工商户“两证整合”登记制度。启动运行市民之家，完善市县乡村政务服务体系建设，推进权力下放、服务下沉，实现“一号申请、一窗受理、一网通办”。深化农村综合改革，推进“三权分置”〔34〕，加快农村产权交易、不动产登记等改革，开展农村资金互助合作试点。统筹推进财税、国有企业、盐业、价格、教育、文化、医药卫生等各项改革。向创新要动力。开展国家知识产权示范城市创建，推动科技引领发展。强化企业创新主体地位，引导企业加大研发投入，推进科技成果转化和产业化，新增高新技术企业50家，高新技术产业增加值增长12％。深入实施“槐荫人才计划”，新增高技能人才3000人。向创业要潜力。深化国家级创业型城市创建，开展全民创业行动，举办“双创活动周”、创业大赛、创业明星评比活动。继续实施“大学生创业资助专项计划”和“回归创业工程”，支持创业大学、创业服务中心等载体建设，扶持2500人就地就近创业。

（五）全力推进项目投资夯实底盘。强力推进重大项目建设。滚动实施100个重大工程项目，力争投资过亿元的项目500个以上，全社会固定资产投资增长16%。实施五大专项行动〔35〕，着力推进十大工业、交通、城建、农业、民生项目。坚持向上争资、向外融资“双促进”，在灾后重建、基础设施等方面争取中省更大支持。快速推进44个灾后重建项目，完成投资73亿元。完善项目建设保障机制。建立常态化项目督查和分级分类调度机制，强化项目审批服务和要素保障，打通项目快速落地绿色通道。最大限度放宽准入门槛，发挥政府资金引导民间投资的作用，拓宽项目融资渠道，加快推进4个部级PPP项目落地建设。扎实开展精准招商、清单招商。落实领导带头招商，集中力量定点、定向、“一对一”跟踪对接招商，及时准确捕捉招商信息，抓好重大项目谋划包装推介，加大与央企、省企合作力度，建立招商项目责任清单，加强反向论证，提高招商引资的针对性和成功率，引进投资过亿元项目75个、过10亿元项目12个。

（六）全力推进城乡统筹协调发展。强化规划引领。认真执行《孝感市城乡规划条例》，推行“多规合一”，健全规委会决策审批、规划督察、监管执法和约谈制度，确保城乡规划从严控管、落地实施。加快城镇建设。全面完成国家新型城镇化试点“4+2”任务〔36〕，确保通过检查验收。孝感城区完成市政设施投资100亿元以上，延伸长征南路、北京南路、玉泉南路，拉通天仙南路北路、董永北路，全面打通城区“断头路”和微循环路网，建立智慧出行平台，缓解交通拥堵问题；完善市高新区、临空区路网体系，改造提标污水处理厂，加快推进垃圾处理静脉产业园，启动建设航空公园、理丝公园二期、董湖公园；推动槐荫生态新区、临空区海绵城市示范区建设，开工10公里地下综合管廊，开通云孝公交。支持县市城区提升基础设施配套水平，争创一批国家和省级特色小镇、特色村。加强精细管理。理顺城市管理执法体制，推进综合执法，下沉执法重心，强化街道、社区管理职责，全面完成背街小巷环境整治，持续推进“四城同创”〔37〕，深化“六大秩序”整治，推进清扫保洁、园林绿化市场化运作，申报全国文明城市提名资格城市和国家卫生城市。推进城乡一体。鼓励市直“三区”和各县市区按照党代会确定的发展定位，错位发展、竞相发展，不断壮大区域经济实力。深入开展新农村“一线一点”建设，启动新一轮三年行动计划，持续开展农村生活垃圾综合治理，建设30个省级、100个市级美丽乡村。

（七）全力推进生态环境绿色宜居。切实加强环境保护。坚持生态优先、绿色发展，开展“绿满澴川”行动，加强天然林保护，争创省级森林城市。开展湖库拆围，退田、退塘、退房还湖16.9万亩，申报临空凤凰国家湿地公园，扩大水域、湿地生态空间。发展绿色交通，倡导绿色出行，实现城区主干道公交站点500米全覆盖。支持县市创建园林城市和卫生城市，开展省级和国家级生态文明建设示范市创建。全面治理环境污染。建立生态文明建设目标评价考核制度，健全完善污染防治考核问责办法和生态环境补偿机制，实行环境保护责任离任审计。启动第二次污染源普查，严格环境监管执法，全面打响大气、水、土壤污染防治三大攻坚战，全面整改落实中省环保督察交办的问题。深入实施大气污染防治行动计划，持续开展重点行业减排、秸杆禁烧和综合利用、扬尘专项整治，确保空气质量优良天数稳定增加。治污水、净湖水、保河水，全面实行河长、湖长、库长负责制，全市工业集聚区污水集中处理设施全部建成并投入使用，今明两年实现乡镇污水处理全覆盖，加快管网配套、污水截流、雨污分流改造，严格保护饮用水源，限期治理水质不达标考核断面；全面规划、分段治理老澴河，城市水体实现“三无”目标〔38〕。全面开展土壤污染详查工作，实施分类防治和管理，推进畜禽养殖污染综合整治，推广农业循环生产技术，治理农业面源污染。节约集约利用资源。强化用地标准管控和供地率考核，盘活存量低效用地，开展国土资源节约集约示范创建，单位GDP地耗下降率6.5%。积极推广绿色建筑，实施重点节能工程，鼓励节能技术改造，加快国家低碳和循环化改造园区试点建设，单位GDP能耗下降3.5%。

（八）全力推进社会事业共建共享。促进教育公平。动工建设东城区中小学，加快老城区学校撤并改造步伐，完成188所学校“全面改薄”任务，建设505套农村教师周转房，加快建设湖北工程技师学院，推动湖北工程学院与湖北职业技术学院联合培养技术技能型人才，实施乡村教师支持计划和教师队伍能力提升工程，资助贫困生6.5万人。优化健康服务。完成3个市级、9个县级卫生计生项目，新改扩建15个乡镇卫生院，启动东城区市儿童医院、疾病预防控制中心建设。全面推行分级诊疗，完善基本药物制度和跨区域医疗报账制度，启动孝感城区公立医院改革。推进“两控四改”〔39〕，90%的常见病患者医疗负担低于20%，实现基本生育免费服务。推动文体繁荣。市文化中心完成布展并对外开放，办好中华孝文化旅游节。推进全民阅读，鼓励文艺精品创作。完成古民居保护立法，加强文化遗产保护。大力开展群众性文体活动，举办演出1800场，安装全民健身器材500套，基本实现农民体育健身工程全覆盖。支持民族宗教、外事侨务、对台工作，扎实做好国防动员、人防、编制、统计、档案、史志、保密、驻外等工作。

（九）全力推进民生改善社会稳定。积极扩大就业。加强公共就业服务信息化和均等化建设，基本建成市县乡就业信息服务体系，就业培训2.8万人次，帮扶困难群体就业8000人，城镇新增就业4.5万人。完善社保体系。全面启动第三代社保卡发卡国家试点，完成机关事业单位养老保险改革，全面推开被征地农民养老保险，建立城乡统一、一体化管理的医疗保险制度，“五项社保”参保突破870万人次。提高社会救助、医疗救助标准，建成60个城市社区红十字服务站，建设市救灾物资储备库，实现农村福利院安全管理“三个系统”全覆盖。维护社会稳定。全面实施“七五”普法规划，推动全民尊法学法守法用法。加强食品药品全程监管，健全完善“红黑名单”制度，整治规范小摊贩、小餐饮、小作坊。建成国家质检中心，开展产品质量全流程监测，保障产品质量安全。推进“互联网+安全生产”行动，深化“两化”体系建设，构建安全监管长效机制。推进基层综治中心规范化，开展“五大民生警务”〔40〕活动，平安乡村视联网覆盖50%的村组、社区，严厉打击暴力恐怖、涉黑涉恶、电信诈骗、网络犯罪行为，支持工会、共青团、妇联等群团组织参与社会治理，提升群众“一感两度两率”〔41〕。加强网上信访、责任信访，引导群众依法合理表达诉求、解决纠纷。加快应急平台建设，加强网络舆情引导，提升应急处置能力。

（十）全力推进政府自身建设。讲政治，忠诚履职。坚持正确的政治方向，牢固树立“四个意识”〔42〕，坚决维护习近平同志的领导核心地位，对党忠诚，言行一致。加快建设学习型政府，自觉用习近平总书记系列重要讲话精神和治国理政新理念新思想新战略武装头脑、指导实践，破除思维定势和工作惯性，提高综合决策、创造性抓落实的能力。牢记为人民服务的根本宗旨，坚决把党中央的大政方针、省委省政府和市委的决策部署，落实到行动上、体现在工作中，用实际成效诠释对党忠诚、展现为民情怀。讲法治，依法履职。认真实施《孝感市法治政府建设实施方案》，把政府工作全面纳入法治轨道，运用法治思维和法治方式化解矛盾、解决问题、推动工作。坚持依法科学民主决策，加强新型智库建设，健全重大决策责任倒查、终身追责机制。自觉接受市人大及其常委会的法律监督和工作监督、市政协的民主监督、各民主党派和社会各界的监督，高质量办理人大代表建议、政协委员提案。讲担当，主动履职。把务实重行、敢于担当作为政府工作的基本要求，以“马上办”的作风干事、以“钉钉子”的韧劲落实。强化对标管理和层级担当，对重点工作、重要改革、重大工程，实行履责清单管理，提高执行力、落实力，确保重难点工作按时间节点推进。加强督查督办，跟踪问效问责，坚决整肃庸政、懒政、怠政，坚决纠正不作为、慢作为、乱作为，确保重大决策部署及时落地生效。健全激励机制和纠错容错机制，鼓励担当、宽容失败，旗帜鲜明地为担当者担当、为负责者负责，充分调动政府工作人员干事创业的积极性、主动性、创造性。讲廉洁，干净履职。坚决落实全面从严治党主体责任,强化“一岗双责”〔43〕，把廉洁从政贯穿政府工作各个环节。用大数据加强对行政权力的监督制约，建立财政收支、国有资产经营、政府采购、公共资源交易、重大工程建设、扶贫资金使用等大数据监管体系，实现对权力集中部门和重点岗位全流程管理，堵住权力寻租空间。强化监督执纪问责，坚决惩治侵害群众利益的不正之风和腐败行为。

各位代表，蓝图已经绘就，扬帆正当其时。让我们紧密团结在以习近平同志为核心的党中央周围，在省委、省政府和市委的坚强领导下，务实重行，真抓实干，奋力推进孝感第三次创业，为全面建成小康社会而努力奋斗！
